# MicroRNAs Bioinformatics Analyses Identifying HDAC Pathway as a Putative Target for Existing Anti‐COVID‐19 Therapeutics

**DOI:** 10.3389/fphar.2020.582003

**Published:** 2020-12-08

**Authors:** Laura Teodori, Piero Sestili, Valeria Madiai, Sofia Coppari, Daniele Fraternale, Marco Bruno Luigi Rocchi, Seeram Ramakrishna, Maria Cristina Albertini

**Affiliations:** ^1^ Diagnostics and Metrology Laboratory, FSN-TECFIS-DIM, ENEA Frascati, Roma, Italy; ^2^ Department of Biomolecular Sciences, University of Urbino Carlo Bo, Urbino, Italy; ^3^ Center for Nanofibers and Nanotechnology, National University of Singapore, Singapore, Singapore

**Keywords:** COVID-19, SARS-CoV-2, HDAC, hypertension, ACE2, off‐label drugs

## Abstract

Over 313,000 SARS-CoV-2 positive cases have been confirmed in Italy as of 30 September 2020, and the number of deaths exceeding thirty-five thousand makes Italy among the list of most significantly affected countries in the world. Such an enormous occurrence of infections and death raises the urgent demand for effective available treatments. Discovering the cellular/molecular mechanisms of SARS-CoV-2 pathogenicity is of paramount importance to understand how the infection becomes a disease and how to plan any therapeutic approach. In this regard, we performed an *in silico* analysis to predict the putative virus targets and evidence the already available therapeutics. Literature experimental results identified angiotensin-converting enzyme ACE and Spike proteins particularly involved in COVID-19. Consequently, we investigated the signalling pathways modulated by the two proteins through query miRNet, the platform linking miRNAs, targets, and functions. Our bioinformatics analysis predicted microRNAs (miRs), miR-335-5p and miR-26b-5p, as being modulated by Spike and ACE together with histone deacetylate (HDAC) pathway. Notably, our results identified ACE/ACE2-ATR1-Cholesterol-HDAC axis signals that also matched with some available clinical data. We hypothesize that the current and EMA-approved, SARS-CoV-2 off-label HDAC inhibitors (HDACis) drugs may be repurposed to limit or block host-virus interactions. Moreover, a ranked list of compounds is provided for further evaluation for safety, efficacy, and effectiveness.

## Introduction

The Coronavirus superfamily includes several human pathogens with large RNA-encoded genomes classified into alpha-, beta- and gamma-coronavirus families. The family’s rate of new virulent human pathogens has increased over the past years, and members of this family have since been identified, which include SARS-CoV (2003), HCoV NL63 (2004), HKU1 (2005), MERS-CoV (2012), and SARS-CoV-2 (previously named 2019-nCoV, 2019). Most of these are reported to involve severe respiratory tract infections. Transmission of SARS-CoV-2 has been reported in humans via respiratory droplets and close interactions. For this reason, social distancing is regarded among key measures. Symptoms usually occur within 2–12 days after infection, although this is still under study. Early symptoms are usually non-specific, the most common being fever, malaise, and myalgia. Other alterations evidenced via laboratory analyses include thrombocytopenia, elevated C-reactive protein, lymphopenia, and elevated lactate dehydrogenase (LDH). Older age, underlying co-morbidities, obesity and smoking habits are the risk factors for the progression to the most severe COVID-19 presentation (Siddiqi and Mehra, 2020; Zhou et al., 2020). Vaccines or direct antiviral drugs are not yet available for COVID-19 infection treatments, but therapeutic etiotropic approaches possibly useful for COVID-19 include: molecules binding to the virus; molecules or inhibitors that target specific enzymes involved in viral docking, replication and transcription; small-molecule inhibitors that target helicase, essential proteases, or other virus proteins; host cell protease or endocytosis inhibitors, siRNA, anti-sense RNA and ribozyme; neutralizing antibodies; monoclonal antibodies (mAbs) targeting host receptors (Kahn and McIntosh, 2005; Caly et al., 2020; Sanders et al., 2020; Wang et al., 2020). Recent publications have brought attention to the possible benefit of chloroquine and hydroxychloroquine, broadly used antimalarial drugs. *In vitro* studies demonstrated their potential efficacy to treat novel coronavirus infection (Vincent et al., 2005). The mechanism underlying the antiviral effect of these latter drugs resides in the abundance of extra nitrogens: once they cross the membrane and enters an organelle, the organelle is prevented from reaching a lower pH, an event which disables the hydrolysis required for coronavirus replication. Alongside this mechanism, chloroquine has also been reported to cause an under-glycosylation of ACE2. Low glycosylation levels of ACE2 strongly reduce the binding affinity of SARS-CoV-2 and consequently its cellular entry. Unfortunately, Randomized Controlled Trials (RCTs) showed that the treatment with hydroxychloroquine provides no benefits in COVID-19 patients (Ortolani and Pastorello, 2020).

Many attempts for developing drugs, and SARS-CoV-2 vaccines, target the spike glycoprotein (S-protein). The viral capsid S-protein is essential for both host specificity and viral infectivity. The S-protein has two subunits, S1 and S2. The S1 subunit receptor-binding domain (RBD) interacts with its host cell receptor, angiotensin-converting enzyme 2 (ACE2), whereas the S2 subunit mediates fusion between the virus and host cell membranes releasing viral RNA into the cytoplasm for replication (Du et al., 2009). The interaction between ACE2 and S-protein is the “armed wing” and the target of possible therapeutic strategies. Non-etiotropic, host-directed drugs include corticosteroids, NSAIDs (Non-Steroidal Anti-Inflammatory Drugs) and low molecular weight heparin.

We focused on discovering putative signaling pathways deregulated by Spike-ACE interaction to repurpose available and approved drugs so as to restore the deregulated pathways during COVID-19 treatment (even natural-based products) (Du et al., 2009; Kumar et al., 2013; Lu, 2020). Many clinical and preclinical anti-SARS-CoV-2 agents are in phase III trials, e.g., remdesivir, oseltamivir, ASC09F (HIV protease inhibitor), lopinavir, ritonavir, darunavir, and cobicistat alone or with interferon-β, convalescent plasma, and monoclonal antibodies (Li and De Clercq, 2020). However, safety and clinical efficacy for COVID-19 cures are not yet available. Vaccines against the disease are on the way, but still unavailable. For this reason, much emphasis has been placed on drug repurposing research for COVID-19 therapy.

Focusing on this topic, we performed an *in silico* analysis using the miRNet platform (Fan et al., 2016). MiRNet is an integrated platform linking microRNAs (miRNAs), targets and functions. Via the integration of multiple, high-quality data sources on miRNA-target interactions and advanced statistical methods within a network visualization system, miRNet allows for browsing through interactions, to obtain significant insight (Fan et al., 2016).

MiRNAs are a class of small non-coding RNAs that mainly act as gene expression negative regulators by binding to 3′-UTR regions of their target protein-coding mRNAs (Baek et al., 2008). Different studies, however, show that miRNAs regulation involves a more complex post-transcriptional control, both repressing and activating gene expression. Groups of miRNAs can induce regulation of specific biological processes, coordinately acting on pathways of functionally related genes (Oliveira et al., 2019). Employing our bioinformatics analyses and available clinical data, we hypothesize a mechanism used by SARS-CoV-2 to infect cells. There are several drugs already approved for different pathologies that can contrast the mechanism we have discovered. This work will facilitate and draw the attention of clinicians to a list of European Medicines Agency (EMA) approved drugs in order to accelerate the selection of the best potential options to fight and contain this pandemic.

## Materials and Methods

### Data Collection

For data collection, a literature search was conducted on PubMed, Web of Science and Scopus with the following key words: ACE; ACE2; AT1R; HDAC inhibitors; hypertension; SARS-COV-2; COVID-19.

The latest public health information from the Centers of disease control (CDC) and the latest research from National Institute of Health websites were also checked. Hence, we were able to find quality clinical data linked to full-text content related to patients infected by COVID-19.

### MiRNet Analyses

MiRNet is an integrated platform linking miRNAs, targets, and functions. We used this easy-to-use web-based tool to investigate the genes modulated by pathways and microRNAs. As indicated in the miRNet website, detailed implementation resources for miRNA-target data are collected from four well-annotated databases, miRTarBase v7.0, TarBase v7.0 and miRecords. This tool offers “statistical, visual and network-based approaches to help researchers understand miRNAs functions and regulatory mechanisms. miRNet offers a comprehensive tool suite to enable statistical analysis and functional interpretation of various data generated from current miRNA studies (Fan et al., 2016).

We performed 3 different analyses to build miRNA-target interaction networks by using 3 input types (either human genes or microRNAs) in the query list: i) ACE and spike; ii) ACE or AT1R, ACE2 and spike; iii) hsa-miR-335-5p and hsa-miR-26b-5p.

### Statistical Analyses

Proportions of severity and proportions of Primary End Point in Hypertension vs patients with Coexisting diseases and in Hypertension vs any Co-existing diseases were compared by chi-square test (Guan et al., 2020). Significance level was fixed at *α* = 0.05.

## Results

### HDACS Deacetylate Histones Pathway Modulated by Coronavirus

**FIGURE 1:**
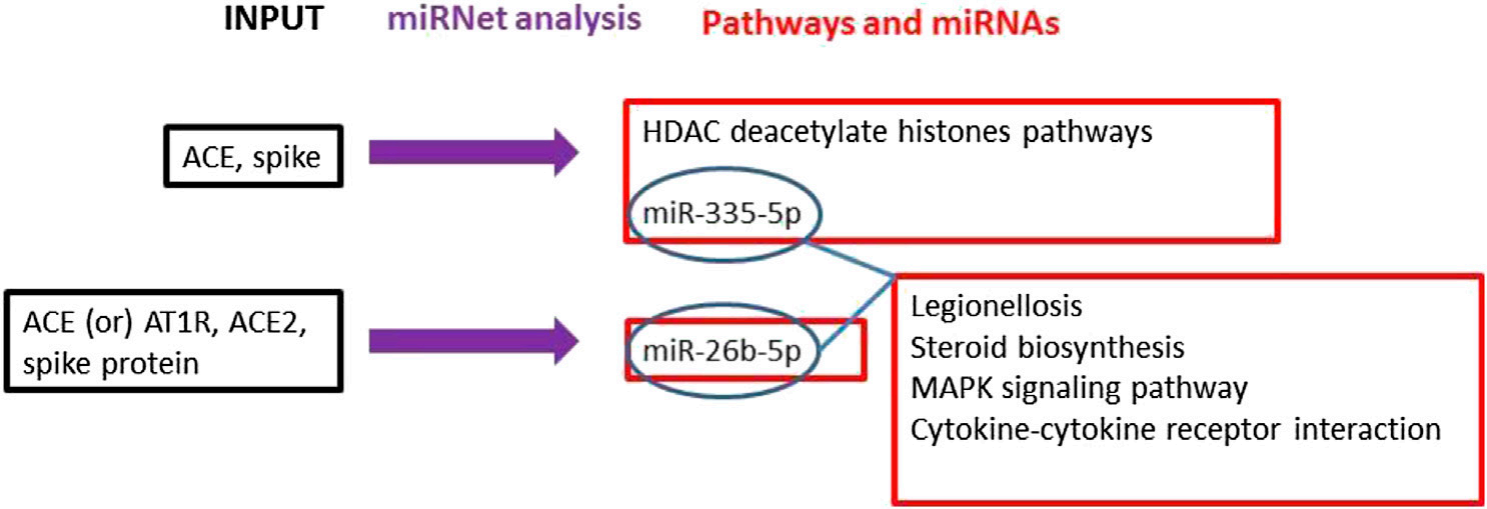
The main mechanisms and interactions surged from our bioinformatics analyses connecting the viral spike protein and ACE2 host receptor are indicated. Workflow (black = input genes used in the analysis; blue = miRNet analysis; red = results obtained by the miRNet analyses).

The literature search on PubMed, Web of Science, and Scopus demonstrated a robust interaction between the virus S-protein and ACE as a host receptor (Li et al., 2005). The main mechanisms and interactions surged from miRNet bioinformatics analysis connecting viral S-protein and ACE2 host receptor returned Histone Deacetylate (HDAC) pathway modulation strongly significant (*p* = 0.0113) with miR-335-5p involvement in ACE and Spike gene expressions. Furthermore, ACE, AT1R and ACE2 miRNet query returned miR-26b-5p modulation as further information (Figure 1).

From the above bioinformatics results, HDAC has a pivotal role in modulating COVID-19 pathogenicity. In detail, it is known that ACE cleaves angiotensin I to generate angiotensin II, whereas ACE2 converts angiotensin II in the vasodilator angiotensin thus funtioning as a negative regulator of the renin-angiotensin (RAS) system (Imai et al., 2005). HDAC acts as a key up-regulator of ACE2 expression by binding its promoter (El Baba and Herbein, 2020). It has also been demonstrated that miR-335 is epigenetically regulated by HDACs (Liew et al., 2020). Histone acetyl-transferases (HATs) acetylates conserved lysine amino acids on histone proteins via the transfer of an acetyl group from acetyl-CoA. Once the acetyl groups, added by HATs to the histones, enter the nucleus, they are removed by HDACs and are incorporated into chromatin. This restores the deacetylated form and, at the same time, releases acetyl-CoA which might be used by 3-hydroxy-3-methyl-glutaryl-coenzyme A (HMG-CoA) reductase. The latter is the key enzyme of cholesterol biosynthesis pathway; notably, plasma membrane cholesterol promotes the binding of COVID-19 to ACE2, fostering virus entry into the cells.

**FIGURE 2:**
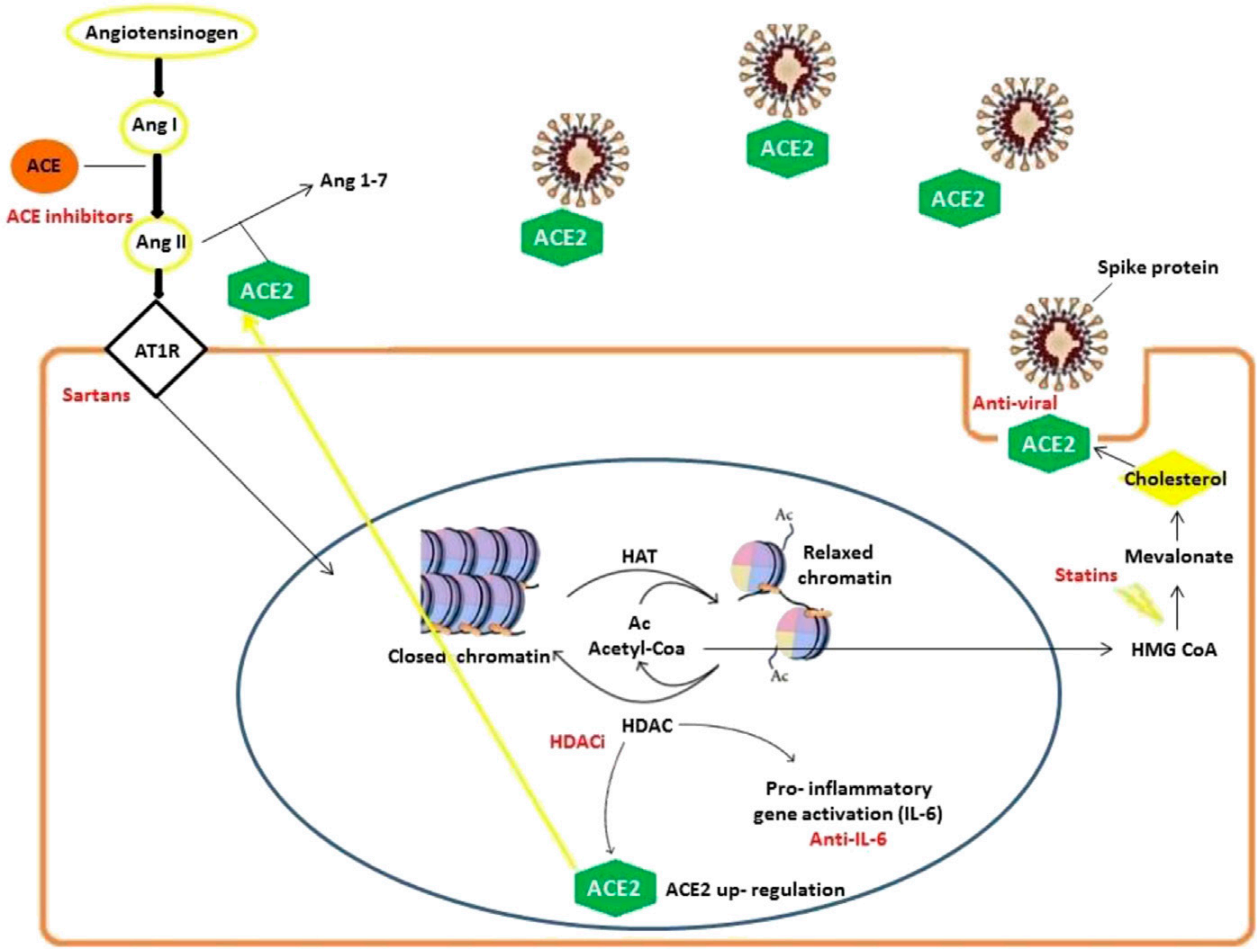
Mechanisms involved in SARS-CoV-2 infection delineating an ACE/ACE2-ATR1-Cholesterol-HDAC axis. SARS-CoV-2 infection occurs with ACE2 and S-protein interactions (facilitated by membrane cholesterol) reducing ACE2 availability. For this reason, excessive angiotensin II (ANG II) accumulates and then activates AT1R (Angiotensin II receptor type 1). The latter in turn stimulates HDAC to up-regulate ACE2 expression and synthesis facilitating SARS-CoV-2 infection. Angiotensin II and AT1R activity increase HDAC function activating inflammatory responses with relevant pathogenetic implications. During HDAC activation, histones are deacetylated and the increased availability of regenerated Acetyl-CoA can feed cholesterol synthesis.

We identified ACE/ACE2-ATR1-Cholesterol-HDAC axis signals that can be affected by SARS-COV-2 infection. In Figure 2 we illustrated the main mechanisms and interactions surged from our bioinformatics analysis connecting viral S-protein and ACE2 host receptor to HDAC activity.

Furthermore, the analysis on miRNet, using miR-335-5p and miR-26b-5p to look for their common modulated pathways, evidenced that four pathways were significantly modulated by both miRNAs: Legionellosis; Steroid biosynthesis; MAPK signalling pathway; Cytokine-cytokine receptor interaction.

### Clinical Comparison: Hypertension in COVID-19 Patients

The mechanism evidenced above suggests that anti-hypertensive drugs acting on renin-angiotensin-aldosterone system (namely ACE-inhibitors and ATR1 antagonists) may interfere with SARS-CoV-2 infection. For this reason, the comparison with clinical data is very important. Indeed, hypertensive patients are treated with these drugs which may interfere with the COVID-19 clinical course by modulating the mechanisms evidenced above (ACE/ACE2-ATR1-Cholesterol-HDAC axis). Hypertensive patients undergoing ACE-inhibitors or ATR1 antagonists, have decreased Angiotensin II production or down-regulated AT1R. In both cases HDACs are not stimulated to produce ACE2, thus limiting its availability for SARS-CoV-2 infection.

To support this hypothesis, we thus analyzed the data retrieved from a Chinese clinical study (Guan et al., 2020). In this study, 1099 patients with laboratory-confirmed COVID-19 from hospitals in China (through January 29, 2020) were analyzed. Primary End Points (admission to an Intensive Care Unit, ICU; use of mechanical ventilation; or death) and severity of the disease have been evaluated. Coexisting illness were also considered: chronic obstructive pulmonary disease (COPD), Diabetes, Coronary heart disease, Cerebrovascular disease, Hepatitis B infection, Cancer, Chronic renal disease and Immunodeficiency. The authors considered 165 hypertensive patients (124/75% without disease severity and 41/25% with disease severity), 261 without any coexisting illness (194/74% without disease severity and 67/26% with disease severity) and 178 with coexisting illness (123/69% without disease severity and 55/31% with disease severity). In addition, among these 165 hypertensive patients, 141/85% were without End Point and 24/15% with End Point; among the 261 patients without any coexisting illness 222/85% were without End Point and 39/15% with End Point; and 139/78% out of the 178 with coexisting illness were without End Point and 39/22% with End Point. These results indicate that patients with hypertension and those without coexisting illness have the same severity and End Points. On the other hand, patients with other illness had higher severity and End Points. In particular, COPD patients were the ones with the highest End Points (58%).

The coexisting illnesses considered in the cited study were COPD, Diabetes, Coronary heart disease, Cerebrovascular disease, Hepatitis B infection, Cancer, Chronic renal disease, and Immunodeficiency.

We performed a comparison by chi-square test to evaluate the proportions of severity and proportions of Primary End Point in Hypertension *vs* patients with Coexisting diseases and in Hypertension vs any Coexisting diseases. As indicated in Table 1, hypertensive SARS-CoV-2 infected patients were mainly without disease severity and without Primary End Points. Specifically, as compared to all the other patients of the study (Non hypertension), hypertensive/infected patients show the same severity primary End Point values as compared to patients without any coexisting disease (data are not significantly different). Furthermore, a favorable trend can be observed when Hypertension is related to the patients with other coexisting diseases, since hypertensive patients demonstrated a higher number of patients with no primary End Point. Also, hypertensive patients related to non-hypertensive/infected patients showed a better disease severity and decreased primary End Point. These results are in favor of our hypothesis that infected COVID-19 hypertensive patients can better support the infection than the other patients with co-existing diseases, an effect which could be plausibly related to the routine anti-hypertensive drug use. Also, patients with hypertension are in line with the disease outcome of infected patients without coexisting illness, suggesting that anti-hypertensive drugs likely generate physiological conditions similar to those of patients that were healthy before COVID-19 infection.

**TABLE 1:** Chi-square analysis for the evaluation of the proportions of severity and proportions of Primary End Point in COVID-19 infected patients.

	Without disease severity	With disease severity	*p* value*
Hypertension	124	41	
Non hypertension	802	132	<0.001
Any coexisting disease	194	67	0.849
Coexisting disease	123	55	0.212
	Primary end point	No primary end point	*p* value*
Hypertension	24	141	
Non hypertension	43	891	<0.001
Any coexisting disease	39	222	0.910
Coexisting disease	39	139	0.078

*Comparison with hypertension (chi-square test).

#### Therapeutics for COVID-19

Six months after its spreading worldwide, COVID-19 is still a difficult malady to treat when it progresses toward its more complicated forms which develop in 10–15% of swab-positive cases, with a very high prevalence in specific at-risk population subgroups. Despite the efforts to identify effective etiotropic drugs, no available agents proved to possess valuable anti-SARS-CoV-2 activity. Important clinical results have been obtained by repositioning host-directed drugs such as, in particular, anti-inflammatory drugs such as corticosteroids, anticoagulants and antiplatelet medications (Kronbichler et al., 2020). However, none of these drugs seems to represent a real “game changer” in COVID-19 treatment, and new drugs are still actively being investigated. To this end, we propose herein to focus on a heterogeneous class of agents which, thanks to their interactions with HDAC circuitries, might offer new chances against COVID-19.

#### HDAC inhibitors as promising Therapeutics for COVID-19 infection

HDACs are enzymes that remove the acetyl group from nuclei histone proteins. In this way, the DNA is less accessible to transcription factors. HDAC inhibitors (HDACi) are a recent class of therapeutic targets in autoimmune and neoplastic diseases (Lin et al., 2006; Nijhuis et al., 2019). These enzymes are able to epigenetically modulate significant cellular functions, altering transcription and also modulating alternative post-translational lysine modifications like methylation, ubiquitination, and sumoylation, by removing acetyl groups from proteins *ε*-amino lysines (Seto and Yoshida, 2014). Abnormal HDACs have an important part in many human pathologies. Other than the use in oncology, it has been reported that HDAC inhibitors may yield a bronchodilator effect, increasing acetylation of substrates other than histones (e.g., HSP20, cortactin) (Comer et al., 2015). Accelerated Phase-2 clinical trials have been approved in Italy and in USA to evaluate the efficacy of tocilzumab in COVID-19 pneumonia patients. Three hundred and thirty patients are enrolled in each of the studies, whose primary end point is to determine the one-month mortality rate (Perrone et al., 2020). A novel mechanism for statins through abrogation of the HDAC activity has also been suggested. Therefore, statins have been proposed as novel HDAC inhibitors for cancer therapy and chemoprevention (Lin et al., 2008).

A list of some EMA/FDA approved inhibitors to be considered for COVID-19, which are already used to cure other diseases, and other HDAC inhibitors have been considered in the clinical trials available on the ClinicalTrials.gov web site (Tables 2 and 3).

**TABLE 2:** EMA/FDA approved HDAC inhibitors.

Classification	Drug	HDAC specificity	Biologic event	Trial stage
*Aliphatic fatty Acids*	Valproic acid	Classes I, II a	Class-switch DNA recombination (CSR) and plasma cell differentiation; CD20 expression	Phase I and II trial
				FDA approved for epilepsy, bipolar disorder and migraine
*Hydroxamate*	SAHA (Vorinostat)	Classes I, II, and IV	CD20 expression; enhances apoptosis mediated by kinase inhibitors that affect BCR signaling and gene expression disruption in Mantle cell lymphoma	FDA approved for cutaneous T cell lymphoma (2006)
	PXD101 (Belinostat)	Pan iHDAC	Cell-cycle arrest or apoptosis of cancerous cells	EMA approved for the treatment of patients with relapsed or refractory peripheral T-cell lymphoma (2014)
	LBH589 (Panobinostat)	Classes I, II, and IV	Reduced cell number and viability; delayed division progression; decreases the number of CD138+ antibody-secreting cells	EMA approved for use and FDA accelerated approval for use In multiple myeloma (in combination with Bortezomib and dexamethasone) (2015)
*Cyclic peptides*	FK228 (Romidepsin)	HDACs 1, 2	Reduced cell number and viability and viability	FDA approved for cutaneous T cell lymphoma (2009) and for peripheral T cell lymphoma (2011)

**TABLE 3:** HDAC inhibitors (still on trial).

Classification	Drug	HDAC specificity	Biologic event	Trial stage
Aliphatic fatty acids	Butirate	Classes I, II a	Apoptosis, cell growth inhibition, cell cycle arrest, and cell differentiation	Phase I and II trial
	AN-9 (Pivanex)	Classes I, II	Apoptosis, differentiation and reduced BCR-ABL protein levels	Phase II and III trials, schizophrenia (NCT02654405; NCT03010865)*
Hydroxamate	ITF2357 (Givinostat)	Classes I, II	Cell proliferation inhibition and apoptosis induction in chronic myelogenous leukemia, BCR-ABL1-positive and childhood B acute lymphoblastic leukemia	Phase II trial Duchenne muscular dystrophy
	4SC201 (Resminostat)	Pan iHDAC	Inhibits proliferation of a large variety of rodent and human cancer cell lines	Phase II trial; advanced stage mycosis fungoides (NCT02953301)*
				Phase II trial for relapsed or refractory Hodgkin's Lymphoma(NCT01037478)*
				Phase 2 hepatocellular carcinoma (NCT00943449)*
	PCI24781(Abexinostat)	Classes I, II	Induces caspase and reactive oxygen species-dependent apoptosis through NF-kappa B mechanisms	Phase I–II trials for B-cell lymphom; (NCT01027910)*
	LAQ-824 (Dacinostat)	Pan iHDAC	Decreases viability in B-ALL, multiple myeloma, and B lymphoma cells	No trials registered
	TSA (Trichostatin A)	Class I and II	CD20 expression (Raji cells)	No trials registered
			Dose-dependent proliferation inhibition (CLBL-1 cells)	
	ACY-241 (Cytarinostat)	HDACs 1, 2, 3, and 6	Inhibition of plainhisma cell myeloma proliferation and survival; cell cycle disruption	Phase I trial; multiple myeloma (NCT02886065)*
	ACY-738	HDACs 1, 2, 3, and 6	Pre-B cell growth inhibition in lupus disease	No trials registered
	Acy-1215 (Riconilostat)	HDAC 6	CD20 expression	Phase II trial; diabetic neuropathic pain (NCT03176472)*
	Tubacin	HDAC 6	CD20 expression; dose-dependent proliferation inhibition	No trials registered
	BML-281	HDAC 6	Blocks B cell infiltration in acute colitis	No trials registered
	LMK-235	HDACs 4, 5	Induces apoptosis and BCLA1 overexpression in diffuse large B cell lymphoma	No trials registered
	RGFP966	HDAC 3	Induces apoptosis, decreases Bcl-2 and Bcl-xL expression.	No trials registered
			Myc-mediated miR expression	
Benzamides	MS-275 (Entinostat)	HDACs 1, 3, 4, 6, 8, and 10	Proliferation inhibition and apoptosis induction; decreases cell viability in B-ALL, B-lymphoma, and multiple myeloma cell lines	Phase I and II—Hodgkin’s lymphoma
				Phase III trial—Metastatic lung cancer
				It has been approved in combination with anticancer tumor compounds
	MGCD0103 (Mocetinostat)	Class I and IV	Modulation of immune response	Phase II trial, urothelial carcinoma (NCT02236195)*, metastatic leiomyosarcoma (NCT02303262)*, non-small cell lung cancer (NCT02954991)*
	CI- 994 (Tacedinaline)	Class I	Proliferation inhibition and apoptosis induction	Phase II and III trials, lung cancer (NCT00005093)*
	AR-42	Class I and II	Cell-cycle arrest and apoptosis via both caspase-3-dependent and caspase-3-independent pathways	Phase I trial; renal cell carcinoma or soft tissue sarcoma (NCT02795819)*, acute myeloid leukemia (NCT01798901)*, multiple myeloma (NCT02569320)*
Cyclic peptides	Apicidin	Class I	Cell growth and cell proliferation inhibition	No trials registered
Mercaptoketone	KD5170	1 and 2	Apoptosis	No trials registered
Sirtuins inhibitors	Nicotinamide	All class III	Cell cycle arrest and autophagy	Phase III trial
	Sirtinol	SIRT 1 and 2	Apoptosis and autophagic cell death	Preclinical
	Cambinol	SIRT 1 and 2	Inhibits SIRT1 and 2 by induced hyperacetylation of p53	Preclinical
	EX-527	SIRT 1 and 2	Inhibits SIRT1 by induced hyperacetylation of p53	Preclinical; phase I and II trials

## Discussion

We elaborated, through bioinformatics tools and literature analyses, a mechanism of SARS-CoV-2 and host interaction. We propose this mechanism as a possible target of several current therapies that may contrast the clinical course of COVID-19. The “repurposing” rationale is to intercept already available therapeutics, as well as SARS-CoV-2 off-label drugs, which are able to contrast the pandemic’s clinical outcome. Indeed, due to the particularly high lethality which has occurred in Italy, with over thirty-five thousand deaths as of 30 September 2020 we, as scientists, were much motivated to urgently indicate some possible interventions. The philosophy of our approach is not to provide a detailed discussion of the explored mechanisms, but rather to take advantage of available data to quickly highlight possible therapeutic attempts. We used the data generously shared by the scientific community and our expertise to contribute towards fighting the pandemic and hopefully help clinicians with new and rational therapeutic strategies.

Two hypotheses guided our research: i) HDACi reduces ACE2 production; and ii) ACE2 reduction decreases virus pathogenicity.

Experimental results from the literature identified cellular ACE2 and viral S-proteins as pivotal players in SARS-CoV-2 docking and pathogenicity (Gurwitz, 2020). Our miRNet analysis predicted miR-335-5p and miR-26b-5p as being modulated by S-protein and ACE together with the HDAC pathway, the latter, as the most significant pathway modulated by ACE and S-protein.

Our results evidenced how HDACs contribute to virus pathogenicity in multiple ways: 1) viral COVID-19 infection occurs *via* ACE2 and spike protein interactions (facilitated by membrane cholesterol) l) which induces excessive angiotensin II-AT1R activation and up-regulates ACE2 through HDAC activity; 2) ACE2 increase facilitates SARS-CoV-2 cellular infection; 3) HDAC function increase activates pro-inflammatory responses which may turn into and 4) Acetyl-Coenzyme A accumulating upon HDAC activity is involved in cholesterol synthesis, whose increased availability may facilitate SARS-CoV-2 cellular entry. These mechanisms are modulated by current therapies. HDAC inhibitors have been included in clinical practice, mainly for haematological malignancy; other existing drugs (e.g. valproate) showed HDAC inhibitory effects as well (Hadden and Advani, 2018). Lin et al., 2006 overviewed of the use of HDAC inhibitors in cancer, focusing on HDAC-mediated acetylation of histone and non-histone substrates, HDAC chemical biology, development of a new type of HDAC inhibitors, and the protein acetylation-independent effect on the activation status of signaling kinases (Seto and Yoshida, 2014).

Our bioinformatics analyses demonstrated the first hypothesis “the molecular mechanism elaborated by our research is that HDACi reduces ACE2 production,” we attempted to demonstrate the second hypothesis “ACE2 reduction decreases virus pathogenicity.” Regarding this, we extracted the clinical data from Guan et al. (2020). Notably, hypertensive SARS-CoV-2 infected patients were mainly without disease severity and without Primary End Points. We ascribe a better clinical progression to the fact that hypertensive patients are under anti-ACE or sartans treatments. Notably, treatments that are now underway interact with all mechanisms we elaborated upon. HDAC is a very interesting, versatile, and critical point because it is at the cross of many important pathways used by the SARS-CoV-2. HDAC inhibitors contrast ACE2, contrast cholesterol productions, as statins do, and contrast IL6 production, as anti-arthritic drugs do. Some HDACs also intervene in viral trafficking via deacetylated microtubules, opening another possible intervention target under our examination currently. The list of drugs we presented is all approved for other pathologies. The mechanism we identified seems to be an appealing target to contrast COVID-19. This mechanism involves ACE/ACE2-ATR1-Cholesterol-HDAC axis and the inhibition of this signaling pathway may result in a decrease of SARS-CoV-2 infection. Furthermore, it has already been demonstrated that the binding of S-protein to ACE2 affects the balance of rennin-angiotensin system (RAS) where the activation of angiotensin type-1 receptor (AT1R) leads to exacerbation of severe pneumonia (Sun et al., 2020). For this reason, the RAS system appears to have a central role in SARS-CoV-2 infection and canonical ACE2 pathway links multiple organ damage in COVID-19. Since SARS-CoV-2 induces ACE2 depletion, it leads to a more extensive conversion of Ang-I into Ang-II via ACE, which then binds to ATR1 receptors (Battagello et al., 2020). When ATR1 is stimulated, this latter in turn stimulates HDAC to up-regulate ACE2 expression and synthesis facilitating SARS-CoV-2 infection.

Our miRNet analysis (common pathways to miR-335-5p and miR-26b-5p) also evidenced that four pathways were significantly modulated: Legionellosis; Steroid biosynthesis; MAPK signaling pathway; Cytokine-cytokine receptor interaction. Interestingly, HDAC has a central role in many diseases in which these pathways are involved and is representing an applicable target under investigation for the discovery of new drugs. Histone acetylation plays an important role in the regulation of pro-inflammatory gene expression in *L. pneumophila* infected lung epithelial cells (Schmeck et al., 2008). Indeed, an increase in the number of superinfections from *Legionella* spp. in SARS has been observed (Bradley and Bryan, 2019). The other pathways are all involved in steroids/cholesterol metabolism. In patients with chronic obstructive pulmonary disease (COPD), where HDAC2 activity is impaired, the inflammatory response is often steroid-resistant (Villagra et al., 2007). For this reason, statins may restore the function and expression of depleted HDAC2 via modulating the mevalonate cascade (Matera et al., 2018). In addition, recently it has been demonstrated that miR-335 is epigenetically regulated by HDACs where HDAC inhibitors up-regulate miR-335 expression (as transcriptional regulators) in Atopic Dermatitis. The authors found that belinostat induced significant expression of miR-335 when compared with other HDACIs (Liew et al., 2020).

S-protein of SARS-CoV-2 interacting with the human ACE2 receptor has also been used for docking-based screening to identify small molecules which bind to either the isolated viral S-protein at its host receptor region or to the S-protein-human ACE2 interface. The hypothesis used in the research considered that small molecules might limit viral recognition of host cells and/or disrupt host-virus interactions (top ligands were based on Vina Score). We propose that the compounds emerging from the above analysis could be tested experimentally (Smith and Smith, 2020). Between them, the ones able to modulate HDAC activity could be taken into consideration: Benserazide; Luteolin-monoarabinoside; Quercetol (quercetin); Protirelin; Benserazide; Vidarabine. Luteolin has also been studied as an alternative therapy for hypercholesterolemia and associated cardiovascular diseases (Baskaran et al., 2015). More importantly, Luteolin has been proposed as an anti-COVID-19 agent because of its mast cell stabilizing activity (Sestili and Stocchi, 2020; Theoharides, 2020).

The discovery of HDAC inhibitors has proven to be an important tool for the study of HDAC functions and mechanism of actions. Some of them have been developed and well described with the purpose to be used for haematological malignancies in clinical practice (Hadden and Advani, 2018). Importantly, HDAC inhibitors seem to be effective bronchodilators by enhancing acetylation of substrates other than histones such as HSP20 and cortactin (Comer et al., 2015). Furthermore, some HDAC inhibitors can block ANG II-induced cardiac hypertrophy. Based on animal studies, ANG II infusion increases class I HDAC2 activity in the heart. Class I HDAC inhibitors also attenuate ANG II-induced cardiac fibrosis (Forrester et al., 2018). Apart from their ability to lower cholesterol, statins have been proven to be able to inhibit the activity of HDAC too. Intriguingly, the above effects of statins might also be useful for SARS-CoV-2 pathogenesis (Lin et al., 2008). Another statins effect deserving attention is their capacity to reduce the expression of TLR4 and regulate the TLR4/Myd88 (myeloid differentiation primary response) NF-κB signaling pathway (Bahrami et al., 2018). This pathway, due to its pro-inflammatory relevance, plays an important role in the severity of respiratory virus infections such as SARS-CoV and MERS-CoV. Indeed, the MYD88 gene was observed to be highly induced by SARS-CoV infection (Yuan, 2015). Furthermore, activation of NF-κB downstream of TLR-MYD88 is a hallmark of coronavirus infections, and its inhibition reduced lung infection, significantly increasing mouse survival after SARS-CoV infection (DeDiego et al., 2014). Interestingly, statins do not significantly affect the MYD88 level under normal conditions, but do prevent its increase during hypoxia or after oxidative stress (Bloom et al., 2010), i.e. two conditions occurring during severe respiratory distress. Atorvastatin was also shown to significantly attenuate TLR4-mediated NF-κB activation (Chansrichavala et al., 2009). The combination of these two effects has been proposed to limit the burst of inflammatory cytokines and chemokines characterizing SARS, MERS and SARS-CoV-2 pneumonia (Fedson et al., 2020).

We identified HDAC at the cross of several molecular routes the virus uses to infect the host, making this molecule family one of the most exploitable candidate for drug use and new drug development. This family is composed by a wide number of heterogeneous agents, ranging from selective inhibitors such as vorinostat, to the antiepileptic valproate, to statins and natural compounds such as quercetin, both classes displaying high pleiotropism due to their anti-inflammatory and antioxidant effects. Despite their heterogeneity, all these compounds inhibit HDAC. The value of sharing common denominator might be three-fold: indeed it paves the way to make drug associations which 1) converge toward the same target with additive effects, 2) exert different but coherent actions by virtue of the pleiotropism of some compounds and finally, 3) allow the reduction of the doses of the single combined agents thus limiting their adverse effects (Teodori et al., 2020).

Our intent is to give a useful insight to be used appropriately by the clinicians in these unprecedented times of recent human history. The information presented here may benefit people all around the world and will be helpful in alleviating the damaging health and social effects of the COVID-19 pandemic.

## Data Availability Statement

The raw data supporting the conclusions of this article will be made available by the authors, without undue reservation, to any qualified researcher.

## Author Contributions

LT, PS, and MCA designed the study and wrote the draft of the manuscript. SC, MBLR and DF searched the literature and analyzed data. SR critically reviewed the manuscript. All the authors have made an extensive contribution to the conception, development, and explanation of results. All the authors also approved the manuscript for publication.

## Conflict of Interest

The authors declare that the research was conducted in the absence of any commercial or financial relationships that could be construed as a potential conflict of interest.
